# Nanocarriers for Inner Ear Disease Therapy

**DOI:** 10.3389/fncel.2021.791573

**Published:** 2021-12-03

**Authors:** Xiaoxiang Xu, Jianwei Zheng, Yanze He, Kun Lin, Shuang Li, Ya Zhang, Peng Song, Yuye Zhou, Xiong Chen

**Affiliations:** ^1^Department of Otorhinolaryngology-Head and Neck Surgery, Zhongnan Hospital of Wuhan University, Wuhan, China; ^2^Department of Otorhinolaryngology, Dawu County People's Hospital, Xiaogan, China; ^3^Department of Biliary Pancreatic Surgery, Tongji Hospital, Tongji Medical College, Huazhong University of Science and Technology, Wuhan, China; ^4^Division of Applied Physical Chemistry, Analytical Chemistry, Department of Chemistry, School of Engineering Sciences in Chemistry, Biotechnology and Health, Kungliga Tekniska Högskolan (KTH) Royal Institute of Technology, Stockholm, Sweden; ^5^Key Laboratory of Applied Surface and Colloid Chemistry, Ministry of Education, School of Chemistry and Chemical Engineering, Shaanxi Normal University, Xi'an, China

**Keywords:** nanocarrier, drug delivery system, inner ear disease therapy, soft material nanoparticle, inorganic nanoparticle

## Abstract

Hearing loss is a common disease due to sensory loss caused by the diseases in the inner ear. The development of delivery systems for inner ear disease therapy is important to achieve high efficiency and reduce side effects. Currently, traditional drug delivery systems exhibit the potential to be used for inner ear disease therapy, but there are still some drawbacks. As nanotechnology is developing these years, one of the solutions is to develop nanoparticle-based delivery systems for inner ear disease therapy. Various nanoparticles, such as soft material and inorganic-based nanoparticles, have been designed, tested, and showed controlled delivery of drugs, improved targeting property to specific cells, and reduced systemic side effects. In this review, we summarized recent progress in nanocarriers for inner ear disease therapy. This review provides useful information on developing promising nanocarriers for the efficient treatment of inner ear diseases and for further clinical applications for inner ear disease therapy.

## Introduction

**Figure 1:**
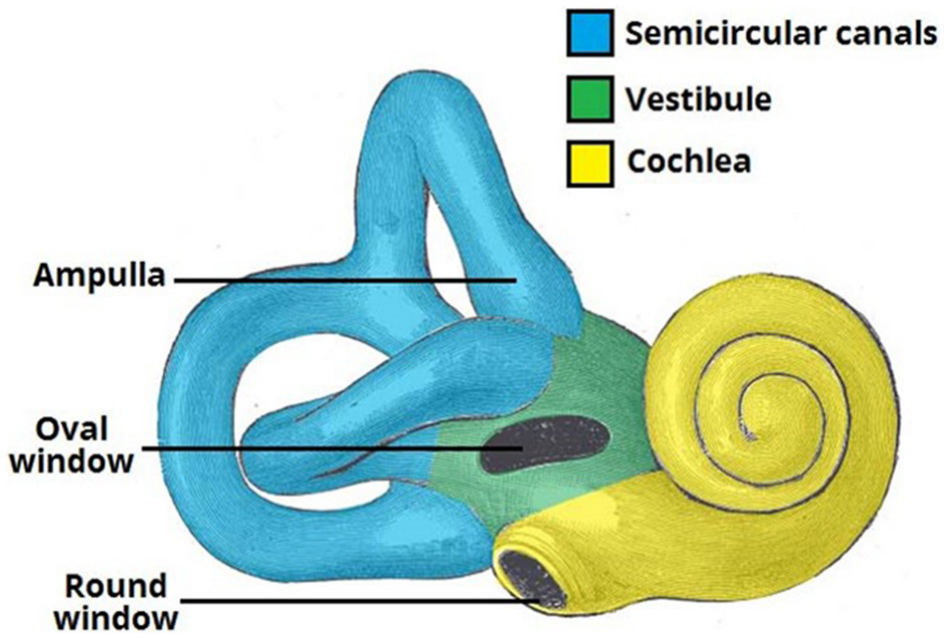
Structure of the inner ear of humans.

Hearing loss is a common disease due to sensory loss that affects human health and life. According to World Health Organization (WHO) data, about 250 million patients suffered from hearing loss in 2005. By 2050, over 5% of the people in the world will suffer from hearing loss (World Health Organization). The production of hearing begins with the collection of sound waves by the outer ear. Then, the sound is transmitted to the hair cells of the inner ear through the middle ear. The inner ear of mammals consists of the vestibule, the semicircular canals, and the cochlea, which is responsible for hearing (Figure 1). The environmental factors, such as excessive acoustic stimulation, aging, infection, autoimmune inner ear diseases, and application of ototoxic drugs, will cause hearing disfunction in the inner ear, directly or indirectly resulting in damage to the cochlear sensory cells and/or related peripheral neurons (Staecker et al., 2001; Ross et al., 2016; Schilder et al., 2019).

The ways to deliver drugs into the inner ear include systemic circulation, from which drugs enter the inner ear through the labyrinth artery, and the round window membrane (RWM). However, current administering drugs have drawbacks, such as disorders, limited labyrinth artery supply, and difficulty in accessing RWM. For example, anti-inflammatory drugs are widely used for inner ear disease therapy; however, the short half-time of drugs in the cochlea that causes rapid elimination is the main problem. Scientists have tried different delivery systems, such as systemic drug delivery systems, intratympanic drug delivery systems, and hydrogel delivery systems, to deliver drugs into the inner ear to treat various inner ear diseases, such as Meniere's disease, autoimmune inner ear disease, and sudden sensorineural hearing loss (SHL) (Havia et al., 2002; Salt, 2005; Nakashima et al., 2016; Rathnam et al., 2019). However, there are drawbacks for each system. It is urgent to develop a new delivery system for high efficiency of inner ear disease therapy, high stability of the drugs before they reach the target cells (outer hair cells as OHC and inner hair cells as IHC) in the inner ear, and the ability to target delivery to the inner ear. Very recently, nanoparticle-based drug delivery systems appeared and attracted the attention of many scientists. Although these systems provide opportunities to solve current problems, there are many things that are unclear and need further investigation. Therefore, a review to summarize recent developments and drawbacks of current nanocarriers for inner ear disease therapy is needed.

In this review, we will summarize the nanocarrier systems for inner ear disease therapy, such as systemic systems, intratympanic systems, hydrogel systems, and nanocarrier systems. Then, we will summarize the soft materials and inorganic nanoparticles that can be used for inner ear disease therapy. Finally, we will conclude the advantages and current challenges of nanocarriers for inner ear disease therapy. This review will provide useful information on nanocarrier drug delivery systems for inner ear disease therapy, and these drug delivery systems could also be further used for other diseases.

## Current Drug Delivery Systems For Inner Ear Disease Therapy

Delivery systems, including systemic drug delivery, intratympanic injection, hydrogel drug delivery, and nanocarrier drug delivery systems, have been used for inner ear disease therapy (Li et al., 2017; Kayyali et al., 2018; Hao and Li, 2019; Mittal et al., 2019; Rathnam et al., 2019; Gheorghe et al., 2021; Jaudoin et al., 2021). Among all current inner ear therapies, the intratympanic injection of liquid drugs is most widely used. Other delivery systems, such as hydrogel delivery systems and nanocarrier delivery systems, are also available. At present, we still need to overcome some barriers.

### Systemic Drug Delivery

Inner ear diseases have been treated by systemic drug delivery systems via the oral route, intramuscular, or intravenous (Ruckenstein, 2004; Alexander et al., 2009; Buniel et al., 2009; McCall et al., 2010; Li et al., 2017). For example, corticosteroids are widely used to treat sudden SHL and have been found to be efficient with a recovery rate of 61%, which is much higher than using a placebo (recovery rate of 32%) demonstrated by Li and Ding (2020). Recently, it was reported that the recovery rates of SHL could reach up to 57–66% with oral corticosteroid (Filipo et al., 2014; Chen et al., 2015). But the limitations of these studies are the small number of patients and the relatively short term for investigation. A long-term course was found when treating autoimmune inner ear disease occurring over weeks to months (Buniel et al., 2009). Nevertheless, when using systemic administration, subtherapeutic local concentrations occur due to the limited blood supply in the inner ear and poor ability to cross the inner ear barrier. However, large doses lead to severe toxicities and undesirable side effects. For example, when aminoglycosides were used, they caused vestibulotoxicity and damage of cochlear, and SHL occurred due to high doses (Graham et al., 1984; Erol, 2007).

### Intratympanic Drug Delivery

Intratympanic systems have also been widely used for inner ear disease therapy. The tympanic membrane is a thin membrane between the external and middle ear. For most of the substances, it is difficult to permeate the tympanic membrane, which is considered a barrier. However, the tympanic membrane is easy to be broken during injection of drugs into the middle ear. Taking steroids as an example, inner ear disease therapy was first treated by intratympanic delivery of steroids in the 1990s (Itoh and Sakata, 1991). At present, intratympanic delivery systems are used for the treatment of sudden sensorineural hearing loss (SHL), Meniere's disease, and vertigo (Chandrasekhar, 2001; Doyle et al., 2004). By using intratympanic delivery systems, the bony structure of the tympanic membrane could be prevented. Moreover, physical barriers, such as the round window membrane, and cellular barriers need to be overcome, when delivering drugs into the inner ear. Small molecules enter the inner ear through the RWM by passive diffusion, which will lead to different concentrations related to the location of the cochlea (Salt and Plontke, 2009). In addition, treatment efficacy during intratympanic injections for inner ear disease therapy will be strongly affected by the parameters, such as cone angle and depth of the tympanic membrane of the patients, and various biological, anatomical, and protocol effects (Volandri et al., 2011).

### Hydrogel Delivery System

To overcome some drawbacks in intratympanic drug delivery systems, such as short residence time and difficulty in sustained release of drugs, hydrogel delivery systems, such as hydrophilic polymeric networks, have been developed for inner ear disease therapy. Chitosan-glycerophosphate hydrogel was developed for the first time to achieve sustained drug release and reduce the variation (Paulson et al., 2008). Chitosan-glycerophosphate hydrogel was a porous matrix and could be degraded by lysozymes to achieve the sustained release of drugs into the inner ear. This hydrogel delivery system showed a low risk of hearing loss and longer vestibular suppression (Xu et al., 2010). In hydrogel delivery systems, drugs are loaded in the hydrogel and located in a certain region, where the drugs can diffuse across the RWM and are released at meaningful concentrations (El Kechai et al., 2015). In contrast, hydrogels exhibit high viscosity and enable a higher residence time of drugs to reach equilibrium in the inner ear. Hydrogel systems could be designed by controlling the chemical composition of monomers and formed by both natural and synthetic products (Li and Mooney, 2016). Therefore, hydrogel delivery systems are potential for inner ear disease therapy due to good biocompatibility, easy functionality, high drug loading, and easy degradation (Ahmed, 2015).

## Nanoparticle-Based Drug Delivery Systems For Inner Ear Disease Therapy

Although the delivery systems mentioned earlier could deliver regulated drugs into the inner ear, they face problems for the delivery of new types of drugs, such as biomolecules. Compared to the regulated drugs, these new types of drugs are less stable within the extracellular compartments. Therefore, it is difficult to deliver new types of drugs to the targeted locations or cells in the inner ear using the delivery systems discussed earlier. Novel drug delivery systems that could overcome these drawbacks are highly demanded in the treatment of inner ear diseases. Recently, various types of nanoparticles, such as nanosized polymer, peptides, silicas, and metal-organic frameworks (MOFs), have been widely employed as drug delivery systems for different kinds of therapies, such as anticancer therapies, anti-inflammatory, and antibacterial therapies. These nanocarriers have a particle size in the nano range, and the particle size could be controlled during the synthesis. For efficient drug delivery, a particle size of <300 nm is usually required to avoid opsonization and elimination (Ulbrich et al., 2016). They also possess a tunable surface with modifiable physicochemical properties for different applications. The nanoparticle-based drug delivery systems could increase the solubility of the drugs, protect the drugs from degradation, prolong the half-life of the drugs during circulation, and allow low passage of the loaded drugs across physiological barriers. Furthermore, these nanocarriers could protect drug properties from degradation, increase the solubility of drugs, difficulty in crossing physiological barriers. Nanoparticle-based delivery systems could also deliver a sustained release of drugs and provide targeted delivery to certain cells. Therefore, nanoparticle-based delivery systems have a great potential for inner ear disease therapy. Nanocarriers for inner ear disease therapy will be discussed in detail in the following section.

### Soft Material Nanoparticle-Based Delivery System

**Figure 2:**
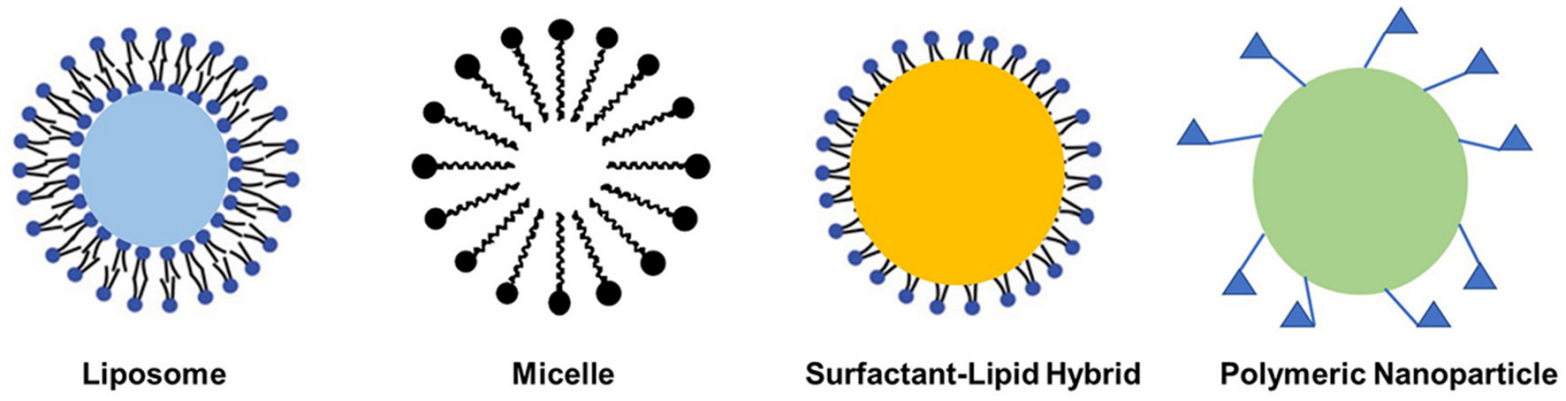
Types of soft material nanoparticle-based delivery systems for inner ear drug delivery.

**Figure 3:**
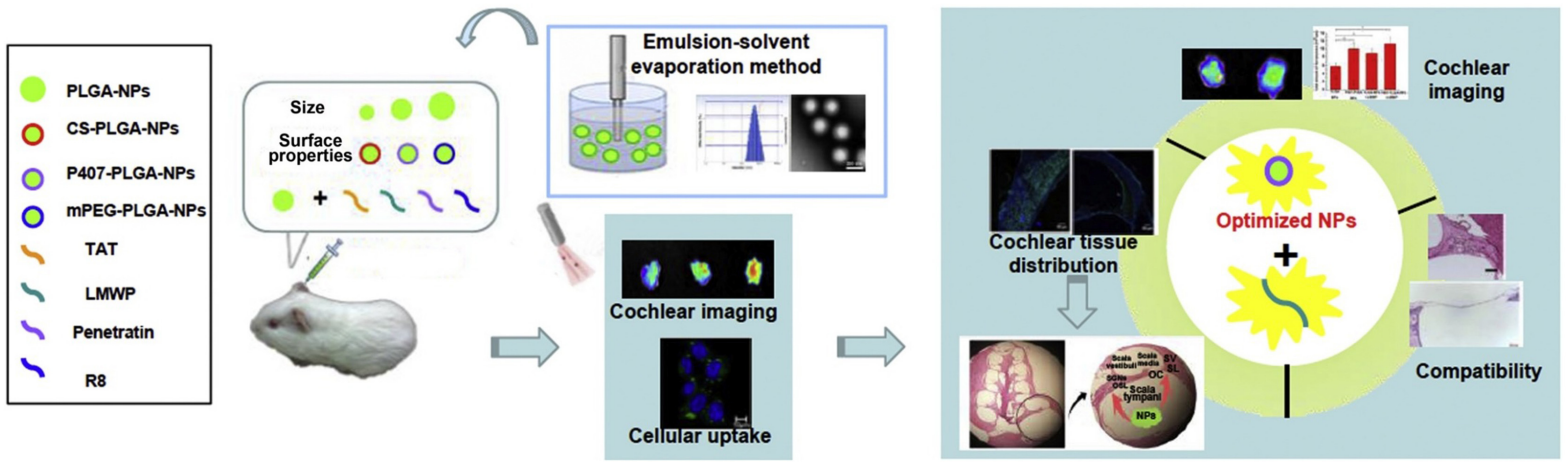
Poly(lactic-*co*-glycolic acid) **(PLGA)** nano-based systems with cell-penetrating peptides for cochlear drug delivery. Reprinted with permission from Cai et al. (2017). Copyright 2017 Published by Elsevier B.V.

Among different nanocarrier delivery systems that can be used for inner ear disease therapy, soft materials, such as polymeric, liposome, micelles, and lipid nanoparticle-based delivery systems, are widely used to load different kinds of drugs (Figure 2; Lu et al., 2011). These soft material nanoparticle-based delivery systems could increase the half-life of drugs and achieve sustained or targeted release of drugs. As demonstrated by Food and Drug Administration (FDA), poly(lactic-*co*-glycolic acid) (PLGA) was a biodegradable polymer and can be easily functionalized for target delivery. When being conjugated with rhodamine, the rhodamine-conjugated PLGA nanoparticles were observed in the cochlea of guinea pigs and long-term residence in the liver due to tissue-specific barriers and fast degradation of PLGA nanoparticles (Figure 3; Palao-Suay et al., 2015; Cai et al., 2017; Szeto et al., 2019). Then, scientists tried to decrease the particle size of the nanocarriers to the range of 150–300 nm, which would enhance the entry of nanoparticles into the inner ear. Further functionalization of the surface with pluronic F127 (PEO_106_-PPO_70_-PEO_106_) increased the accumulation of particles (Zou et al., 2010; Leso et al., 2019). PEGylated polymers could increase the half-life, biocompatibility, and solubility of the loaded drugs and decrease the side effects, as well as the immune reactions, to drugs (Veronese and Mero, 2008). The selected location of nanoparticles in the inner ear was also achieved by modifying the nanoparticles with chitosan, which changed the surface charge and hydrophilicity of the nanoparticles. These modifications could help nanoparticles reach the inner ear before endocytosis. Other types of polymeric nanoparticles, such as dendriplexes and chitosan-based nanoparticles, poly(l-lactic acid) (PLLA), PLLA-PEG, polycaprolactone (PCL), and polyethylene glycol (PEG), have also been used for inner ear disease therapy to encapsulate drugs via electrostatic interaction or hydrophobic-hydrophobic interaction (Dash et al., 2011; Wang et al., 2011; El Kechai et al., 2015; Lajud et al., 2015; Vigani et al., 2019).

Micelles and liposomes are formed by molecules with both hydrophobic and hydrophilic parts. Micelles have a hydrophobic environment inside micelles and enable the loading and delivery of hydrophobic drugs encapsulated inside, while the hydrophilic property of the outer surface of micelles increases their solubilization in aqueous solutions. Furthermore, micelle-based delivery systems could also protect unstable drugs from biological attacks during circulation. When liposome nanoparticles are used as delivery systems for inner ear disease therapy, both the external and internal surface of the liposome are hydrophilic with the same structure as the phospholipid bilayer (Panahi et al., 2017; Zylberberg et al., 2017). Amphiphilic liposomes can carry them across the RWM and deliver them into the cells (Uri et al., 2003; Meyer et al., 2012). Liposomes degrade readily in cells, resulting in low toxicity of liposomal drugs. Multifunctional liposome nanoparticles could be prepared by modifying the surface with polyethylene glycol, carbohydrates, and folic acid.

There are many other soft materials of nanometer size that could also be used for inner ear disease therapy. Cubosomes formed by a lipid core with a single lipid bilayer and a polymeric shell were very efficient for loading drugs (Barriga et al., 2019). Solid lipid nanoparticles and lipid nanocapsules have also been reported (Gao et al., 2015; Yang et al., 2018). Protein nanoparticles are another option to increase the delivery to the tissue and reduce the toxicity of compounds (Lohcharoenkal et al., 2014).

### Inorganic Nanoparticle-Based Delivery System

**Figure 4:**
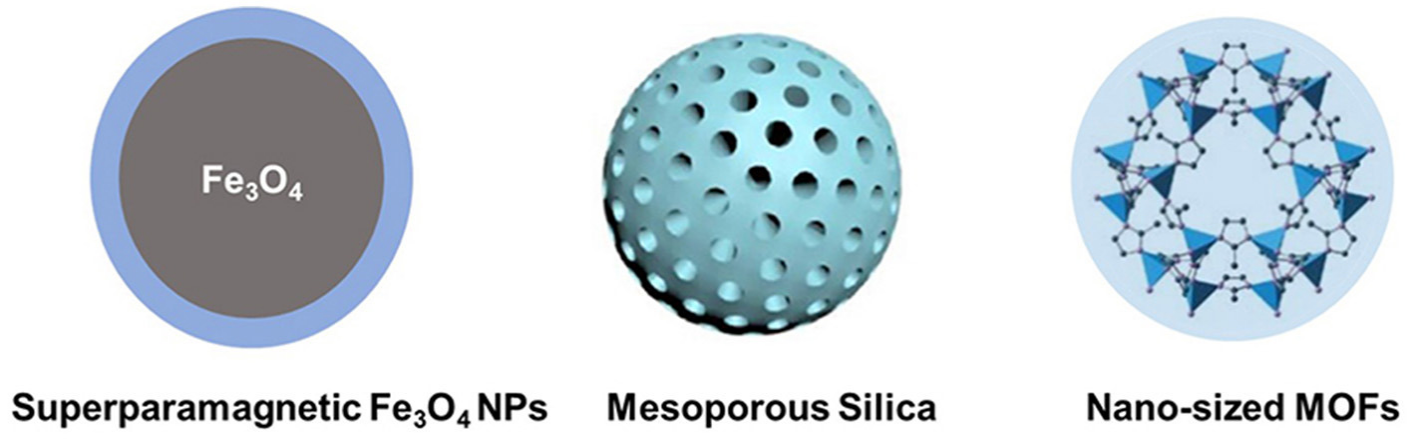
Types of inorganic nanoparticle-based delivery systems for inner ear drug delivery.

Compared to soft materials, inorganic nanoparticle-based delivery systems are still in the developing stage. Inorganic nanoparticle-based delivery systems could provide unique properties, such as antimicrobial and magnetic properties (Figure 4). Although inorganic nanoparticles provide opportunities for inner ear disease therapy due to some useful qualities, there are many drawbacks, such as high price and limited biological stability.

Superparamagnetic Fe_3_O_4_ nanoparticles are a kind of inorganic material. These materials could pass the round window by magnetic force and arrive at the inner ear (Guigou et al., 2021). Superparamagnetic iron oxide nanoparticles are easy to synthesize and exhibit low toxicity and intrinsic antimicrobial activity for effective delivery systems (Rodrigues et al., 2019). Superparamagnetic iron oxide nanoparticles have no pores to encapsulate drug molecules, while in combination with polymers, such as PLGA and chitosan, superparamagnetic iron oxide nanoparticles could adsorb drugs via the electrostatic/hydrophobic interaction for inner ear drug delivery (Grumezescu et al., 2013; Sangaiya and Jayaprakash, 2018). Other metal-oxide-based nanoparticles have shown effective inner ear disease therapy-related infectious diseases. For example, TiO_2_ nanoparticles are photosensitive and show activity against fungi and bacteria (Luksiene, 2017). Zinc oxide, copper oxide, calcium oxide, silver oxide, aluminum oxide, and zirconium oxide have also been widely investigated as carriers for biomedical applications (Narayanan et al., 2012; Karimiyan et al., 2015; Swaminathan and Sharma, 2019). However, the particle size of these metal oxides is still difficult to control, and the target delivery systems using these metal oxides are needed to be designed to specifically locate nanoparticles in the inner ear.

Porous nanoparticles, such as mesoporous silica nanoparticles, and MOF nanoparticles possess pores for the encapsulation of both hydrophobic and hydrophilic drugs. These materials are usually easy to prepare, have large pore volumes, and can be easily functionalized. Mesoporous silica nanoparticles are biocompatible and could slowly degrade under physiological conditions (Zheng et al., 2011, 2013; Bernardos et al., 2019). These materials can be used to construct “smart” delivery systems, which are sensitive to changes in the environment, including changes to the pH, thermal, and magnetic fields. Recently, mesoporous silica nanoparticles could be used to control the encapsulation and release of antibiotics (Selvarajan et al., 2020). Furthermore, the surface of porous silica nanoparticles could be functionalized to target the designed tissues and used for inner ear disease therapy (Tang et al., 2012). When loaded with a brain-derived neurotrophic factor, porous silica nanoparticles could target the spiral ganglion neurons and release drugs for a long time period (Schmidt et al., 2018). More recently, MOFs have been designed and studied for many applications, including biomedical applications (Zheng et al., 2016; Kaneti et al., 2017; Wu and Yang, 2017; Lu et al., 2018). Recently, Xu et al. (2020) encapsulated methylprednisolone (MP) in ZIF-90 nanoparticles for the treatment of inner ear disease for the first time. ZIF-90 prevents the degradation of drugs during circulation after intraperitoneal injection and delivers MP into the inner ear. These MOF nanoparticles exhibit good protection from noise, low damage to the inner ear structure, and low nephrotoxicity during therapy.

Metallic and metal oxide nanoparticles are one group of the most promising inorganic materials for inner ear disease therapy (Paladini et al., 2015). Silver (Ag) nanoparticles could interact with the surface of bacterial cells and break the cell membranes to achieve permeability (Hamad et al., 2020). Ag nanoparticles could enter the inner ear after intraperitoneal injection and break pathogens with antibiotic formulations, which could overcome the drawbacks and achieve high efficacy in the ear therapy (Muhsin and Hachim, 2014; Zou et al., 2015). Similar situation happens to gold (Au) nanoparticles, which are used for loading drugs and imaging applications. Au nanoparticles have shown potential to be used as inner ear contrast agents and are located in cochlear cells (Kayyali et al., 2017). However, at present, Ag and Au nanoparticles have not yet been used for inner ear disease therapy. Quantum dots, such as semiconductor nanocrystals, show unique optical properties and are considered another option (Liu et al., 2010). Due to their advantages of good biocompatibility, biodistribution, stability, and long half-life, metallic and metal oxide nanoparticles and their combinations with polymers and/or proteins are highly potential for inner ear disease therapy in the near future.

### Targeting Modification of Nanoparticle-Based Delivery System

Nanoparticles could increase circulation time and prevent the degradation of drugs. Moreover, a specific surface modification could further enable the nanoparticle-based drug delivery systems with target properties to reach a specific type of cell, which is ideal for inner ear therapies to deliver the drugs to a specific type of inner ear cell. Ligands have a specific interaction with certain cells and, thus, could be used for cell-specific targeting of nanoparticles for inner ear disease therapy (Frutos et al., 2018; Valero et al., 2018). For example, in an *in vitro* model prepared from the mouse cochleae, peptide-based nanoparticles could interact with spiral ganglion cells through tyrosine kinase and p75 neurotrophin receptors (Roy et al., 2010). Cy3-labeled silica nanoparticles were demonstrated to be located within the inner ear of RWM of mice compared to the control group of mice (Praetorius et al., 2007). Cy3-labeled silica nanoparticles could also reach central auditory nuclei and superior olive through retrograde axon transport. To target OHCs, prestin was connected to peptides and coupled to nanoparticles. The cellular uptake of these nanoparticles in the OHCs of rat cochleae was achieved (Surovtseva et al., 2012). Using a similar method, nanoparticles were found to be taken up by OHCs *in vivo* (Wang et al., 2018). Using this method, the nanoparticles were located into designed cells, and the uptake efficiency could also be improved.

Moreover, a deep understanding of a suitable ligand for targeting delivery is needed. The interaction between ligand-loaded nanoparticles, and the receptor in the inner ear should be further investigated. The targeting delivery system is also interesting and with high potential to be developed for inner ear disease therapy (Li et al., 2017).

## Conclusion And Prospects

In this review, we summarized the currently used systems for inner ear disease therapy, including the most widely used delivery systems (i.e., systemic, intratympanic, and hydrogel delivery systems) and nanotechnology-based systems. To overcome the barriers of these systems in overcoming barriers in the inner ear, nanoparticle-based drug delivery systems, which have shown many advantages in the treatment of various diseases, have gained increasing attention in inner ear diseases during the past years. Several soft materials and inorganic-based nanoparticles have been investigated and shown to improve the efficiency of drugs, enhance antimicrobial performance, and reduce the side effects of inner ear disease therapy. Nanoparticles could cross the barrier in the inner ear, deliver drugs into the inner ear with low side effects, and remain harmless for healthy tissues. Furthermore, we discussed the targeting drug delivery systems using nanoparticles by modifying the surface with ligands, proteins, and so on. Developing multifunctional nanoparticles that could target specific cells and release drugs in a controlled manner is the way for the future.

Although nanocarriers have been used for inner ear disease therapy, there are still many works that need to be carried out in the future. First, the information on the interaction between nanoparticles and ear toxicity, such as effects on organs, is still unclear. The long-term investigation of health effects could be studied in the future. Second, the critical physicochemical characteristics that affect their biodistribution and the way to overcome physical and cellular barriers are not well-defined. The rational design of nanoparticles to achieve target delivery to the inner ear needs more effort. In summary, nanoparticle-based delivery systems have brought potential solutions and paved a novel way for inner ear disease therapy, but it still has a long way to go for real clinical applications.

## Author Contributions

XX, JZ, YH, KL, SL, YZha, PS, YZho, and XC prepared this manuscript. YZho and XC guided all aspects of this work. All authors contributed to the article and approved the submitted version.

## Funding

This work was supported by Swedish Research Council (2021-00295).

## Conflict of Interest

The authors declare that the research was conducted in the absence of any commercial or financial relationships that could be construed as a potential conflict of interest.

## Publisher's Note

All claims expressed in this article are solely those of the authors and do not necessarily represent those of their affiliated organizations, or those of the publisher, the editors and the reviewers. Any product that may be evaluated in this article, or claim that may be made by its manufacturer, is not guaranteed or endorsed by the publisher.
